# Flavor compounds in Vine Tea (*Ampelopsis grossedentata*) infusions

**DOI:** 10.1002/fsn3.1754

**Published:** 2020-06-29

**Authors:** Renata C. V. Carneiro, Hengjian Wang, Susan E. Duncan, Sean F. O'Keefe

**Affiliations:** ^1^ Department of Food Science and Technology Virginia Tech Blacksburg VA USA

**Keywords:** *Ampelopsis grossedentata*, flavor, GC‐MS, HS‐SPME, vine tea

## Abstract

Vine tea (*Ampelopsis grossedentata*) is a tea traditionally used in Chinese herbal medicine that is rich in the natural antioxidant dihydromyricetin (ampelopsin). In addition to its multiple health benefits, vine tea extracts and dihydromyricetin have been suggested as potential natural antioxidants for food applications, such as soybean oil and meat products. However, there is still little information available on vine tea chemistry, and in particular the volatile profile and sensory characteristics, which can affect product acceptability and restrict its use as a natural antioxidant. The objective of this exploratory study was to identify potential volatile components present in vine tea in order to support further research and applications in the food industry. Vine tea infusions brewed from commercial samples were characterized by acidic pH values and a dark, reddish‐yellow color. Twenty‐one volatile compounds were identified as potential flavor components of vine tea, including aldehydes and ketones. Further studies are suggested to quantify the volatile compounds and understand their importance to vine tea's aroma profile. Sensory studies are also suggested to access consumer's acceptability of vine tea and products containing vine tea as an ingredient.

## Graphical abstract

This study identified potential volatile components present in vine tea in order to support further research and applications in the food industry.

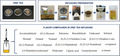

## 1 INTRODUCTION

The interest of commercial, academic and governmental sectors in functional food development has increased over the years. The fast‐growing global tea market and consumers' increasing desire for accessible healthier products, such as tea and tea‐based products, has driven the tea industry to innovate and improve tea variety, availability, quality (e.g., aroma, taste, color), safety, and convenience (Heck & Gonzalez de Mejia, 2009; Jones & Jew, 2007; Tea Association of the United States of America, 2020). Several herbal teas or tisanes have been consumed around the world for pleasure, health care, and disease prevention; some popular examples are rooibos (*Aspalathus linearis*), rose hip (*Rosa* spp.), chamomile (*Matricaria recutita*), rosemary (*Rosmarinus officinalis*), fennel (*Foeniculum vulgare* subsp. *vulgare*), lavender (*Lavandula angustifolia*), thyme (*Thymus vulgaris*), maté (*Ilex paraguariensis*), hibiscus (*Hibiscus sabdariffa*), and peppermint (*Mentha piperita*) (Heck & Gonzalez de Mejia, 2009; Lasekan & Lasekan, 2012; da Silva Pinto, 2013; Tschiggerl & Bucar, 2012; Wong, Liang, Chen, & Zhao, 2015). Vine tea (*Ampelopsis grossedentata*) is a healthy herbal tea, rich in the natural antioxidant dihydromyricetin (ampelopsin), and whose dried leaves and stems have been traditionally used in Chinese herbal medicine (Gao et al., 2009; Ye, Wang, Duncan, Eigel, & O'Keefe, 2015; Zheng et al., 2014).

The number of researchers studying vine tea health properties, chemical components, and possible applications has been increasing significantly in the past few years in different broad research areas. In food science, vine tea has been seen beyond a pleasant healthy beverage; vine tea extracts have been suggested as potential natural antioxidants to increase shelf‐life of meat products such as cooked ground beef (Ye et al., 2015) and cooked mixed pork patties (Zhang, Xu, Xue, Jiang, & Liu, 2019). Tschiggerl and Bucar (2012) reported there is a dearth of qualitative and quantitative information of volatile components in herbal tea infusions and the changes of their volatile composition due to tea preparation process. Accordingly, there is still a lack of information about flavor volatile components of vine tea. Overall, acceptability and quality of herbal teas depend on their aroma and flavor quality, so it is important to characterize aroma compounds in teas so evaluation of aroma quality can begin. In addition, flavor components can limit alternative use as a food ingredient. Ye et al. (2015) suggested studies on the sensory characteristics of vine tea extracts and dihydromyricetin were important to evaluate their potential use as food antioxidants. Therefore, the objective of this study was to identify volatile components present in vine tea (*Ampelopsis grossedentata*) flavor profile in order to provide valuable information for further studies and support its application in new food and beverage products.

## 2 MATERIALS AND METHODS

### 2.1 Vine tea samples

Vine tea samples of three commercial brands (A, B, and C) were randomly chosen and purchased from tea stores in Zhangjiajie, China. All samples had vine tea as their only ingredient listed (100% vine tea) and were composed by dried leaves and stems of *Ampelopsis grossedentata*. Preparation of vine tea infusions was adapted from Sheibani et al. (2016). Boiling distilled (DI) water (98°C) was poured over vine tea dried leaves and stems in a ratio of 2 g of tea per 100 ml of water. After brewed for 5 min, vine tea infusions were filtered using gravity filtration technique and qualitative grade plain filter paper circles (diameter 24.0 cm, P4 grade paper, medium‐fine porosity, slow flow rate; Fisher Scientific). All laboratory glassware was washed, rinsed with distilled (DI) water, and baked overnight at 160°C before use. Vine tea samples were brewed and analyzed in triplicate.

### 2.2 Characterization of vine tea infusions: pH and color measurement

A Fisher Scientific™ Accumet™ Research AR25 pH/mV/°C/ISE Meter calibrated with buffer solutions (pH 4.0 and 7.0) was used to measure pH of vine tea infusions. A Minolta CR‐300 Chroma Meter calibrated with a standard white plate (L* = 96.77, a* = 0.45, b* = 2.37) was used to measure the reflective color of vine tea infusions in CIE L*a*b* (CIELAB) system. Vine tea infusions (30 ml) were pipetted into glass test tubes which were placed in a special support for liquids (CRA‐70, Minolta Co.). All samples were measured in triplicate. Statistical analyzes were performed using JMP^®^ Pro 11.0.0, and a 5% significance level was used.

### 2.3 Volatile extraction by SPME and GC‐MS analysis

Volatile characterization of vine tea infusions took place at the Virginia Tech Food Analysis Laboratory. Filtered vine tea infusion (10 ml) and 2.0 g of salt (NaCl) were placed into 20 ml amber glass headspace vials (Supelco) with 18 mm magnetic screw thread cap with PTFE/Red Chlorobutyl or PTFE/Silicone septa (Restek). Clean glassware and vials were rinsed with DI water and baked overnight at 160°C before use. Glass vials and screw caps were new and were not reused. Vials were kept in a refrigerated tray (4°C) until before analyzes were performed. Volatile compounds of vine tea infusions were extracted by headspace solid‐phase micro‐extraction (HS‐SPME) and analyzed with gas chromatography‐mass spectrometry (GC‐MS) using the method adapted from Sheibani et al. (2016). Samples were prepared, extracted, and analyzed in triplicates. An AOC‐5000 Plus SPME auto‐sampler (Shimadzu Scientific) was used for sample extraction and injection into the GC‐MS. Samples were equilibrated for 2 min prior to extraction, then a divinylbenzene/carboxen/polydimethylsiloxane (DVB/CAR/PDMS) fiber (50/30 µm, 2 cm length) (Supelco) was exposed to the headspace above vine tea infusions in amber glass vials for 30 min at 40°C with an agitation speed of 250 rpm. Injections were made in splitless mode with injector temperature of 240°C, and fibers were left in the injection port for 5 min to ensure complete desorption.

A Shimadzu GCMS‐QP2010 Ultra gas chromatograph with mass selective detector (Shimadzu Scientific) was used to identify volatile headspace compounds. Volatiles desorbed from the fiber were separated by using a polyethylene glycol capillary column (ZB‐Wax plus; 60 m × 0.25 mm i.d. × 0.25 µm film thickness; Phenomenex) and a DB5 capillary column (SHRXI‐5M Serial # 1007600; 30 m × 0.25 mm i.d. × 0.25 µm film thickness; Shimadzu Scientific, Columbia, MD, USA). Ultra‐high purity helium was used as the carrier gas at a flow rate of 0.5 ml/min and linear velocity of 30 cm/sec. Initial oven temperature was held at 40°C for 0.5 min and increased to 240°C (final temperature) with a temperature ramp rate of 8ºC/min. Mass spectra were obtained every 0.3 s and data collected from m/z 40–400. The ion source and quadrupole temperatures were set to 230 and 200°C, respectively.

Chromatograms were plotted by Shimadzu software, and volatile compounds were first identified by comparison of their mass spectra to fragmentation spectra of standards of the NIST 11 (Scientific Instrument Services) and Wiley 2010 (John Wiley and Sons Inc.) libraries. A series of *n*‐alkanes (C5, C6, C7, C8, C10, C12, C14, C16, C18, C20, C22, C24; Supelco) was used to calculate Linear Retention Index (LRI) using linear regression. Online databases (Flavornet and Pherobase) and peer‐reviewed papers were consulted for confirmative identification of volatile compounds by matching LRI values for both columns, and for understanding of odor descriptions.

## 3 RESULTS AND DISCUSSION

Overall, vine tea infusions were characterized by acid pH values (pH ~ 4.3) and dark, reddish‐yellow color (Table 1). Although all infusions were prepared following the same method (section 2.1), significant differences were observed in their pH and color characteristics. As shown in Table 1, sample C had the most acidic infusion, and samples A and C had significantly darker infusions than sample B, which presented lower L* value. The acidic characteristic of vine tea infusions was similar to the characteristics of black tea infusions (pH = 4.95 when prepared with distilled water [pH = 7]) reported by Liang and Xu (2001). The different pH and color profiles of the vine tea infusions could be attributed to possible differences in quality standards (e.g., proportion of dried leaves and stems), production and/or processing conditions of the commercial tea samples, which were unknown to the researchers (a study limitation). Color is an important quality attribute in tea infusions and it is known that flavonoids have a relationship with plant colors (Chaturvedula & Prakash, 2011; Lin, Yang, Hsieh, Liu, & Mau, 2014; Wang, Park, Chung, Baik, & Park, 2004). Vine tea is known to be rich in flavonoids, which may contribute to its infusions typical color. However, the relationship between color and chemical composition of vine tea infusions was not evaluated and further study is suggested. The pH of vine tea infusions may impact sensory attributes such as color, flavor, and taste. Zhang et al. (2019) reported cooked mixed pork patties treated with vine tea extract (0.1% and 0.3%) had lower pH and L*, a*, and b* values than their control (formulated without antioxidants). Researchers have shown that pH is an important parameter for optimization of the extraction of green tea constituents such as catechins, caffeine, and theanine (Vuong, Golding, Stathopoulos, & Roach, 2013). As vine tea extracts have shown great potential for applications in the food industry, further studies of the impact of brewing solution pH are also suggested to optimize the productions of vine tea extracts and powders.

**Table 1:** pH and color characterization of vine tea infusions

Vine tea	pH	L*	a*	b*
A	4.39 ± 0.03^a^	26.45 ± 0.47^a^	0.87 ± 0.19^a^	6.56 ± 0.16^a^
B	4.38 ± 0.04^a^	28.51 ± 0.76^b^	−0.13 ± 0.39^b^	8.62 ± 0.25^b^
C	4.29 ± 0.01^b^	26.56 ± 0.61^a^	1.45 ± 0.20^c^	7.82 ± 0.69^c^

Note

^a‐d^Means ± *SD* followed by the different letters in the same column are significantly different (*p* < .05).

A total of 21 volatile compounds were identified as potential characteristic components of vine tea flavor profiles (Table 2). As the authors did not have control of quality standards, production, and/or processing parameters of the commercial vine tea samples used in the study, it seemed appropriated to consider repeatability as a relevant indication to suggest a volatile compound as a potential characteristic compound of vine tea flavor profile. Therefore, only volatile compounds that were present in samples of at least two vine tea brands, were identified in both GC‐MS columns (ZB‐Wax and DB5), and had their calculated LRI confirmed with literature were reported in Table 2 as potential components of vine tea flavor profiles. Additional volatile compounds that failed in some of the criteria described above (e.g., present in infusions of only one vine tea commercial sample, or present in the chromatograms of only one GC‐MS column) were not reported. Although sensory characteristics of vine tea volatile compounds were not evaluated and quantified in this study, odor descriptions from available literature were provided in Table 2, as they may be used as reference for future sensory studies (e.g., lexicon development and check‐all‐that‐apply list of descriptors).

**Table 2:** Potential characteristic volatile compounds of vine tea (*Ampelopsis grossedentata*) flavor profile

Number	Volatile compound^a^	Column	LRI	LRI	Odor description (Literature)
			(Calculated)	(Literature)	
1	Isovaleraldehyde (3‐Methylbutanal)	ZB‐Wax	902	912^1^, 990^3^, range: 906–943^5^	Fruity, almond, toasted, malty, green, herbal.^1, 3^
		DB5	675	646^1^, 654^1^, range: 629–669^5^	
2	Hexanal	ZB‐Wax	1,068	1,064^6^, 1,067^1^, 1,081^4^, 1,084^2^, 1,097^3^, range: 1,056–1,106^5^	Green, fruity, acorn, tallowy, fishy, grassy, herbal, leafy, fatty, apple‐like, fresh.^1, 2, 3, 4, 7^
		DB5	801	784^1^, 797^1^, 799^1^, 800^1^, 801^2^, 802^1^, 803^1^, 805^1^, 819^1^, range: 782–810^5^	
3	(E)‐2‐Pentenal	ZB‐Wax	1,114	1,104^6^, 1,117^1^	Pungent, apple, fruity, strawberry, oily, soapy.^1, 7^
		DB5	758	754^1^	
4	(E)‐2‐Hexenal	ZB‐Wax	1,206	1,192^2,6^, 1,224^3^, 1,229^4^, range: 1,196–1,238^5^	Green, leafy.^2, 3^
		DB5	850	844^2^, range: 837–865^5^	
5	Octanal	ZB‐Wax	1,269	1,278^6^, 1,280^2^, 1,300^1^, 1,302^1^, 1,307^1^, range: 1,267–1,312^5^	Lemon, stewed, boiled meat, rancid, soapy, citrus, green, flower, fruity, orange, fatty.^1, 2^
		DB5	1,005	1,006^2^, range: 993–1,012^5^	
6	(Z)‐2‐Heptenal	ZB‐Wax	1,299	1,319^1^, 1,320^1^	—
		DB5	957	964^1^	
7	6‐methyl‐5‐hepten‐2‐one (Sulcatone)	ZB‐Wax	1,309	1,319^1^, 1,320^6^, 1,347^3^, range: 1,317–1,357^5^	Mushroom, earthy, vinyl, rubbery, woody, blackcurrant, boiled fruit, sweet, fruity.^1, 3^
		DB5	989	985^1^, range: 977–995^5^	
8	Nonanal	ZB‐Wax	1,363	1,368^6^, 1,385^2^, 1,397^3^, 1,402^1^, 1,408^1^, 1,415^1^, range: 1,370–1,414^5^	Gravy, green, tallowy, fruity, gas, chlorine, floral, waxy, sweet, melon, soapy, fatty, lavender, citrus fruit, oily.^1, 2, 3^
		DB5	1,108	1,104^2^, range: 1,093–1,118^5^	
9	(E,E)‐2,4‐Heptadienal	ZB‐Wax	1,457	1,456^6^, 1,489^4^, 1,502^3^, range: 1,455–1,514^5^	Fatty, oily.^3^
		DB5	1,014	1,011^2^, range: 996–1,019^5^	
10	Benzaldehyde	ZB‐Wax	1,482	1,500^1^, 1,522^1^, 1,525^1^, 1,495^2^, 1,536^3^, 1,515^4^, 1,482^6^, range: 1,481–1,555^5^	Burnt sugar, almond, woody, fragrant, sweet, almond.^1, 2, 3, 4^
		DB5	964	960^2^, range: 947–982^5^	
11	(E,Z)‐3,5‐Octadien‐2‐one	ZB‐Wax	1,484	1,484^6^	Synthetic, plastic, fatty, fruity^1, 7^
		DB5	1,097	1,096^1^, 1,098^1^	
12	(E,E)‐3,5‐Octadien‐2‐one	ZB‐Wax	1,533	1,521^1^, 1,576^4^, 1,539^6^	Fresh, sweet, woody, mushroom.^1, 7^
		DB5	1,074	1,068^1^	
13	(E,Z)‐2,6‐Nonadienal	ZB‐Wax	1,548	1,597^1^, 1,605^1^, range: 1,555–1,601^5^	Cucumber, melon. ^1, 7^
		DB5	1,157	1,154^2^, range: 1,148–1,162^5^	
14	2,6‐Dimethylcyclohexanol	ZB‐Wax	1,564	1,598^4^	—
		DB5	1,118	1,112^1^	
15	Undecanal	ZB‐Wax	1,569	1,624^1^, range: 1,582–1,630^5^	Fruity, green, waxy, oily.^1^
		DB5	1,313	1,310^1^, range: 1,295–1,319^5^	
16	Safranal	ZB‐Wax	1,607	1,596^1,6^, 1,648^2^	Powerful saffron aroma, tobacco, camphor, herb, sweet. ^1, 2^
		DB5	1,208	1,201^1^	
17	α‐terpineol	ZB‐Wax	1,660	1,662^6^, 1,688^2^, 1,692^3^, 1,695^4^, range: 1,659–1,724^5^	Oily, anise, minty, floral, lilac‐like. ^2, 3^
		DB5	1,197	1,185^1^, 1,195^2^, 1,207^1^, range: 1,178–1,203^5^	
18	Dodecanal	ZB‐Wax	1,678	1,700^1^, 1,718^1^, 1,722^1^, 1,728^1^, range: 1,685–1,732^5^	Oily, herbal, fatty, citrus, waxy.^1^
		DB5	1,414	1,401^1^, 1,407^1^, 1,409^1^, 1,413^1^, range: 1,397–1,420^5^	
19	Caproic acid (Hexanoic acid)	ZB‐Wax	1,796	1,807^6^, 1,829^2^, 1,845^4^, 1,847^1^, 1,863^1^, 1,872^1^, range: 1,807–1,873^5^	Sweaty, pungent, cheesy, goat‐like, rancid.^1, 2^
		DB5	973	Range: 969–1,026^5^	
20	Geranylacetone	ZB‐Wax	1,826	1,840^2^	Fresh, floral, rose, green, fruity, magnolia.^1,2^
		DB5	1,461	1,436^1^, 1,448^1,2^, 1,453^1^	
21	α‐ionone	ZB‐Wax	1,827	1,809^2^, 1,818^6^, 1,842^4^, range: 1,798–1,892^5^	Woody, violet‐like, floral.^2,4^
		DB5	1,442	1,422^2^, range: 1,403–1,435^5^	

Note

References (LRI and odor description): ^1^The Pherobase: Database of pheromones and semiochemicals. ^2^Acree and Arn (2004). ^3^Qin et al. (2013). ^4^Wang et al. (2010). ^5^Babushok, Linstrom, and Zenkevich (2011) ^6^Kawakami et al. (1995). ^7^Venkateshwarlu, Let, Meyer, & Jacobsen (2004).

^a^ Volatile compound was present in infusions of at least two vine tea commercial samples, was identified in both GC‐MS columns (ZB‐Wax and DB5), and had calculated LRI confirmed with literature.

More than a few volatile compounds identified in the vine tea samples are recognized flavor components of nonherbal teas (*Camellia sinensis* L.) and other herbal teas. The aldehydes hexanal (green, grassy, metallic odor), (E,E)‐2,4‐heptadienal (fatty, orange oil, oily odor), and nonanal (fatty, oily odor), for example, were also reported in black, green, and oolong teas (Lee, Chambers, Chambers, Adhikari, & Yoon, 2013; Sheibani et al., 2016; Qin et al., 2013). Hexanal is a fatty acid‐derived volatile resulting from linoleic acid degradation with an associated green odor that also describes other aldehydes, such as octanal and nonanal. Hexanal was also reported in rooibos and cocoa teas and is a key odorant in expresso coffee (Cerny, 2010; Kawakami, Ganguly, Banerjee, & Kobayashi, 1995; Lasekan & Lasekan, 2012; Maeztu et al., 2001; Qin et al., 2013; Wang, Wang, Li, Ye, & Kubota, 2010; Yang, Baldermann, & Watanabe, 2013). Among the aldehydes, (E)‐2‐hexenal (fruity, strawberry, cherry odor) was reported in oolong tea, and (E,E)‐2,4‐heptadienal was reported in cocoa teas (Kawakami et al., 1995; Kumazawa, 2006; Qin et al., 2013; Sheibani et al., 2016; Wang et al., 2010). The volatile component 3‐methylbutanal, also known as isovaleraldehyde, is associated with a malty odor and was previously reported as an odorant of cocoa mass and one of the key odorants of expresso coffee and brews prepared from arabica and robusta coffees (Cheetham, 2002; Maeztu et al., 2001). Safranal, described as powerful saffron aroma, tobacco, camphor, herb, and sweet odor, hexanoic acid (caproic acid), and 2‐ethylfuran were volatile compounds present in vine tea that was also reported in black tea (Kraujalytė, Pelvan, & Alasalvar, 2016). (E)‐2‐pentenal, described in the literature as fruity, strawberry‐like odor, was also found in oolong tea, while (Z)‐2‐heptenal was previously reported in safflower (*Carthamus tinctorius* L.) flower head buds, and leaves, olives, and virgin oil of *Olea europaea* cultivar Olivastra Seggianese (Binder, Benson, & Flath, 1990; Flamini, Cioni, & Morelli, 2003; Kawakami et al., 1995). Some compounds present in vine tea were previously reported as constituents of the volatile profile of fruits, for example, octanal (lemon, stewed, boiled meat, rancid, soapy, citrus, green, flower, fruity, orange, fatty odor) and undecanal (fruity, green, waxy, oily odor) were both present in mango fruits, and (E,Z)‐2,6‐nonadienal (cucumber, melon odor) was reported as an important aroma compound in apricot (Buttery, 1993; Pino, Mesa, Muñoz, Martí, & Marbot, 2005). Dodecanal (oily, herbal, fatty, citrus, waxy) is a component of conventionally prepared and poroplast‐extracted hydrocarbon‐free orange oils, as well as octanal and decanal (Cheetham, 2002).

Benzaldehyde (fragrant, sweet, almond/nutty odor) and 2,6‐dimethylcyclohexanol (odor description not found) are volatile compounds also reported in green tea and cocoa tea infusion; benzaldehyde is a typical component for formulation of natural black cherry flavor, as well as hexanoic acid, and it was reported in black and oolong teas (Cheetham, 2002; Kawakami et al., 1995; Lee, Chambers, Chambers, Adhikari, & Yoon, 2013; Qin et al., 2013; Sheibani et al., 2016; Wang et al., 2010). Geranylacetone (fresh, floral, rose, green, fruity, magnolia odor), α‐ionone (woody odor), and α‐terpineol (oily, anise, minty, floral, lilac‐like odor) are three volatile compounds identified in vine tea that are among the main volatile compounds of mate infusion tea (Lasekan & Lasekan, 2012). Additionally, the alcohol α‐terpineol was also identified as flavor compound in peppermint and rosemary infusions, green mate, jasmine, chamomile, green, oolong and black teas (Kawakami et al., 1995; Lasekan & Lasekan, 2012; Qin et al., 2013; Riachi, Abi‐Zaid, Moreira, & Maria, 2012; Tschiggerl & Bucar, 2010, 2012).

Among the ketones identified in vine tea infusions, α‐ionone, the isomers (E,Z)‐3,5‐octadien‐2‐one (synthetic, plastic odor, fatty, fruity odor) and (E,E)‐3,5‐octadien‐2‐one (fresh, sweet odor), and geranylacetone were similarly identified in oolong tea, and α‐ionone was also present in cocoa tea, black, and green teas (Kawakami et al., 1995; Qin et al., 2013; Sheibani et al., 2016; Wang et al., 2010). In addition, 6‐methyl‐5‐hepten‐2‐one, also known as sulcatone and associated with mushroom, earthy, vinyl, rubbery, woody, blackcurrant, boiled fruit, sweet, fruity odor, was reported as an important volatile component in tomato, as well as the aldehydes (E)‐2‐hexenal and hexanal (Buttery, 1993).

## 4 CONCLUSIONS

Several of the 21 volatile compounds identified as potential characteristic components of vine tea flavor profile are known components of the aroma profile of other teas (nonherbal and herbal) largely consumed worldwide. Differences in volatile profiles observed among the commercial vine tea samples could be associated with unknown differences in quality standards, harvest and/or processing conditions, and the fact that vine tea is still significantly produced from wild plants in China. This exploratory study provides an initial picture of volatile compounds that may contribute to vine tea flavor profile. Additional studies, such as gas chromatography‐olfactometry (GC‐O) and sensory evaluation (descriptive and acceptability studies), are suggested in order to understand the importance of these volatile components to vine tea flavor profile and how they can potentially affect acceptability of products containing vine tea. Moreover, further studies are suggested to be performed using samples from controlled harvest and processing, as quality standards and both production and processing conditions may affect the volatile profile of products from plant sources such as herbal teas.

## CONFLICT OF INTEREST

The authors declare no known conflict of interest.

## AUTHOR CONTRIBUTIONS

RC collected and analyzed the data, and initial draft. All authors critically revised and edited manuscript content. SO approved the final version of the manuscript to be published.

## ETHICAL APPROVAL

This study did not involve any human or animal testing.
